# Supplementary material for: Conformations and cryo-force spectroscopy of spray-deposited single-strand DNA on gold
Source: Nat Commun. 2019 Feb 8;10:685. doi: 10.1038/s41467-019-08531-4 (PMC6368621; doi:10.1038/s41467-019-08531-4)
Supplement: Supplementary file 1 — Supplementary Information [file 41467_2019_8531_MOESM1_ESM.pdf]

Supplementary information:
Conformations and cryo-force spectroscopy of spray-deposited single-strand DNA on gold

Authors: Rémy Pawlak *et al.*

Supplementary Methods

Supplementary Note 1 : ElectroSpray Deposition (ESD).

The MolecularSpray (http://www.molecularspray.co.uk/) apparatus connected to the preparation chamber allows the injection of molecules contained in a solution from a syringe in air to UHV through a capillary and three differential pumping stages separated by leak orifices [1-2]. The spray is form **in** ed by applying a high voltage between the syringe and the grounded inlet capillary (inner diameter 750 µm). This leads to the formation of a jet of multiply charged droplets that are accelerated then pass through the differential pumping system. Solvent evaporation causes coulomb fission which results in smaller droplets with increasing concentration of ssDNA and salt, down to nano-droplets containing single molecules and eventually gas-phase ionized complexes. During ESD, the base pressure of the preparation chamber reaches p = 10^{-7} mbar. Typical high voltage values ∼1.5 kV are adjusted to control the spray quality by visually observing the overall spray shape during deposition. In solution solvated DNA and ions interact via screened electrostatic interactions, and the ions form a neutralizing space-charge sheath around each DNA molecule. This also happens in the interior of large droplets but, in the final nanodroplets, solvation shells may overlap, so that Na^+ counter-ions become strongly bound to nearby PO_4^- ions of the ssDNA backbone. Before deposition these nanodroplets carry surface charges, most likely excess Na^+ ions, as indicated by mass spectra of proteins electro-sprayed from similar solutions by other investigators[2]. Upon impinging on the metallic surface, however, those excess ions are very likely neutralized since the gold substrate is grounded.

To visualize possible conformations of ssDNA electro-sprayed on the Au(111) surface, we systematically recorded constant-current STM images at 4 K. During deposition, the substrate was kept at room temperature then annealed step-by-step as shown in Fig. 2 of the main text, and cooled to 4 K in between. STM images of as received and annealed samples are shown in Supplementary Figures 1a and b. The rare occurrence of small features besides single ssDNA oligomers with dark stripes of the Au(111) herringbone reconstruction clearly observed in Supplementary Figure 1b lead us to conclude that the contamination of the sample by water solvent molecules is well-below a mono-layer coverage.

To activate the desorption of the remaining solvent molecules, we annealed the surface from 340 K to more than 500 K. This promoted the diffusion and self-assembly of ssDNA oligomers as described in the main text. Supplementary Figure 2c shows a cut along the red line in b and Supplementary Figure 2d the frequency shift recorded along the same line using the same tip with a picked up CO molecule at the apex.

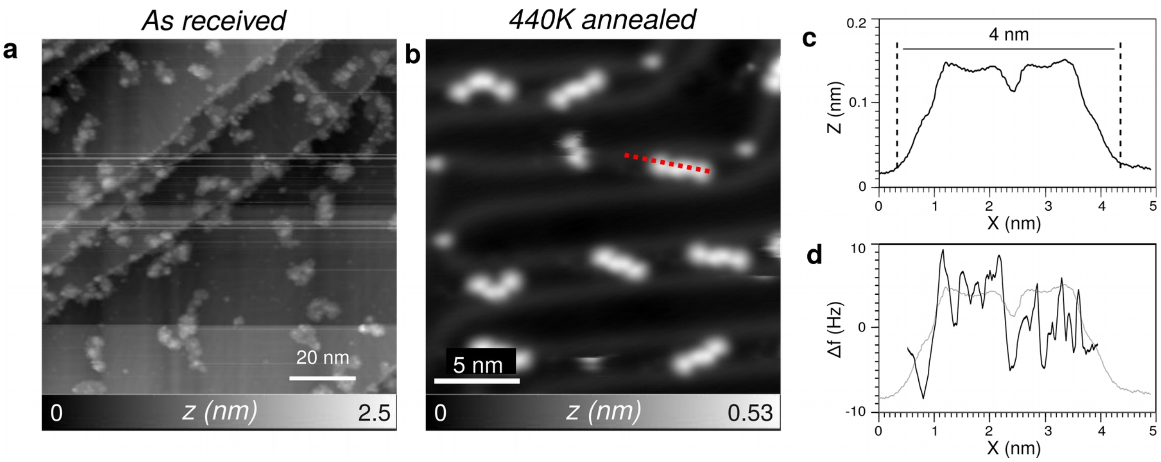

Supplementary Figure 1. Spray-deposited of ssDNA in ultra-high vacuum at room temperature. **a,** STM topographic images of after spray-deposition on the Au(111) kept at room temperature. **b,** STM topographic image after sample annealing at 440K (I = 1 pA, V = 1.2 V). Both STM images were recorded at 4K. **c-d,** Profiles taken along the red dashed line in **b** of a of the topography Z(X) and frequency shift Δf(X).

Supplementary Note 2: DNA structure.

A single DNA consists of a sequence of nucleotide bases (dark-blue hexagons in Supplementary Figure 2), such as adenine, cytosine, guanine and thymine, that are attached to a phosphate backbone (cyan ribbon in Supplementary Figure 2) through a deoxy-ribose sugar (cyan pentagon in Supplementary Figure 2). In nature, DNA is most commonly found as a double-stranded molecule (Fig. 1a) where two single-strands, held together by inter-molecular hydrogen bonds between complementary bases, wrap around each other forming a helical structure. This structure has very well defined parameters which are key features in understanding both mechanical properties of DNA as well as its interaction with proteins. The helical rise corresponds to the distance between consecutive base-pairs along the longitudinal DNA axis. Assuming the same helical rise of ~ 0.33 nm obtained from numerical calculations for the 20-cytosine dsDNA, a single strand would have a total length of ~6.3 nm (Supplementary Figure 2a),

albeit without considering the reduced mechanical stability of ssDNA and the influence of the environment, e.g. of water and/or of the substrate upon adsorption.

Another important property of ssDNA is that each phosphate caries one negative charge which is, however, compensated by ions from the environment. In the case of dsDNA, on average 90 % of the solute cations (Na^+) are accumulated, whereas only 10 % of anions (Cl^-) are depleted within a few nanometers **[5]**.The combined effects of molecular flexibility, electrostatic and other interactions lead to a complex environment-dependent potential energy landscape of ssDNA molecules.

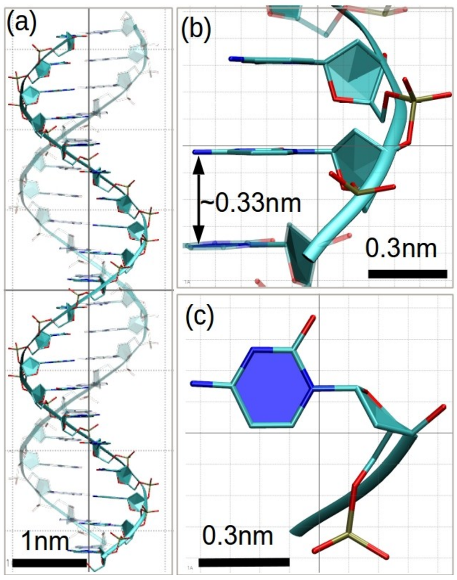

Supplementary Figure 2. Schematic Representation of a single-strand DNA (ssDNA) composed of 20 cytosines. **a,** Side-view of ~~a~~ double stranded DNA (dsDNA) in its canonical B-form. The 20 cytosine-mer used as a starting configuration in simulations is represented using an opaque texture. Its complementary strand (not included in our simulations) is represented using a transparent texture. **b,** Close-up side view showing the helical rise of 0.33 nm. **c,** Top view of a single cytosine base and of one connection to the phosphate backbone. Bonded atoms are represented as bars with the following color scheme: red (oxygen), cyan (carbon), blue (nitrogen), brown (phosphorous). Hydrogens are not represented for clarity.

Supplementary Note 3 : Simulation protocols.

All simulations used in this work are schematically summarized in Supplementary Figure 3. They were performed in constant N and T ensembles where a Langevin thermostat mantained the average temperatures specified in Supplementary Figure 3. Stage 3b, stage 4 stage 5 and stage 6 simulations were performed in vacuum. In all the others water molecules were considered as specified below. In all simulations we consider one 20-cytosine ssDNA oligomer and include 19 Na^+ counter-ions to ensure overall charge neutrality.

Stage 1: 20-cytosine ssDNA equilibrated in aqueous solution. Here we start from a 20-cytosine DNA single strand in the canonical B-form generated using the NAB software available in AMBERTOOLS put it in the center of a 11.6x11.6x11.6nm^3 simulation box filled with 41190 water molecules and 19 sodium counter-ions. After an energy minimization to avoid steric clashes in the MD simulation we performed a 100 ns long NPT run to equilibrate the pressure to 1 atm and the temperature to 300 K (using a Langevin thermostat coupled to all atoms and a Berendsen barostat coupled to the box walls). Results are discussed in Supplementary Note 4.

Stage 2: Equilibration of a water droplet containing a ssDNA oligomer in vacuum at 300 K. The atomic configuration is obtained from the last nanoseconds of stage 1 by keeping only water molecules within 1~nm from the ssDNA (composing its 3 hydration layers and totaling 1332 water molecules) and the 19 sodium counter-ions. Then we placed this selection inside a empty 10x10x10 nm^3 simulation box much larger than the system size. Following an initial energy minimization, we performed a NVT simulation at 300 K during 100 ns. Results are discussed in Supplementary Note 4. Thus stages 1 and 2 allowed us to generate a stable charge-compensated hydrated ssDNA oligomer inside a nano-droplet in vacuum.

Stage 3: Adsorption of a water droplet containing a ssDNA molecule onto a Au(111) surface at 300 K. In this stage we position the above-mentioned nano-droplet 2 nm above a 16x16nm^2 Au(111) three layer slab and let the droplet freely adsorb onto the surface while performing NVT simulation at a constant temperature of 300 K in a 16.7 nm high box largely surpassing the system dimensions. Results are discussed in the main text.

We subsequently used the final configuration of stage 3 to study the influence of water removal as described in the following two stages.

Stage 3a: Water evaporation at 450 K from a hydrated ssDNA molecule adsorbed on Au(111). Starting from the final configuration obtained in stage 3 we annealed the system at 450 K during 100 ns in the NVT ensemble.

Stage 3b: Equilibration at 400 K of a fully dehydrated ssDNA molecule adsorbed on Au(111). Instead of pursuing the former simulation, we again started from the final configuration obtained in stage 3 but removed all water molecules, i.e. kept only the adsorbed cytosine 20-mer, the 19 Na^+ counter-ions and the same

Au(111) slab. Then we performed a 100 ns long annealing MD simulation in the NVT ensemble at 400 K.

Results from stages 3a and 3b, as well as stage 4 are discussed and compared in Supplementary Note 5.

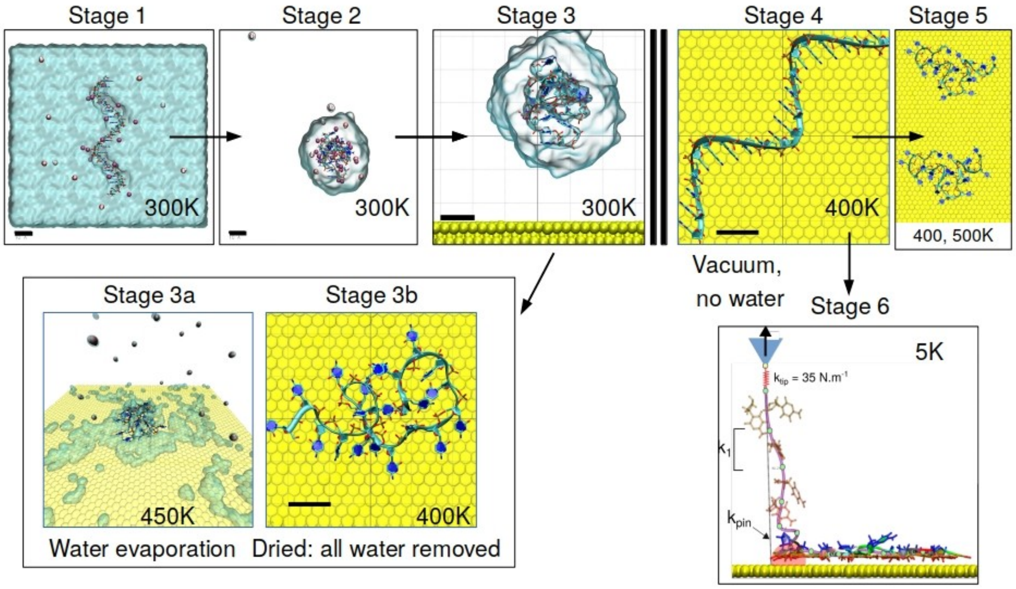

Supplementary Figure 3. Chart describing the different MD simulations performed and their relationship. The arrows indicate that the final configuration of a given stage is used as a starting configuration of the connected stage, e.g. the final configuration of stage 3 is used as a starting configuration of stages 3a and 3b. Gold atoms are represented as yellow van der Waals (VdW) spheres; ssDNA as liquorice; transparent cyan Connolly surfaces enclose the water molecules present in the system; and purple VdW spheres represent sodium counter-ions.

Stage 4: Adsorption of a ssDNA molecule onto Au(111) at 400 K in vacuum. In order to check whether adsorption in vacuum would lead to a different configuration, we started from a completely different configuration. A single 20-mer strand in the canonical B-form (see stage 4 in Supplementary Figure 3) was placed 1 nm above the same 16x16 nm^2 Au(111) slab and 19 Na^+ counter-ions were positioned by tleap AMBER tool in between the phosphates. Then we let the system evolve in a 100 ns long MD simulation performed in the NVT ensemble at 400 K. We also started from different relative orientation of the ssDNA strand with respect to the surface (up-right, inclined 45°) and obtained final ssDNA configurations quite similar to those in all adsorption simulations discussed here (stage3, stage3a, stage3b, stage4) and in Supplementary Note 5,

i.e. compact folded ssDNA conformations ~2 nm wide and with a ~4 nm overall length, as shown in Fig.1c of the main text.

Stage 5: Temperature-assisted self-assembly of two ssDNA molecules. On top of a 18x22 nm^2 Au(111) three layer slab we place two ssDNA molecules with a lateral separation of 3nm. The atomic coordinates of each ssDNAs molecule and of the attached counter-ions are obtained from the final configuration of stage 4. We let the system evolve in a NVT ensemble at 400 K and 500 K during 500 ns in order to visualize which internal and relative rearrangements occur upon annealing in vacuum. Results are discussed in the main text and in the Supplementary Note 6.

Stage 6: Lifting a ssDNA molecule adsorbed on Au(111). As a starting configuration we use that obtained at the end of stage 4 (in vacuum and with the corresponding counter-ions). Then, via a steered MD simulation at 5K one end of the molecule is lifted along the direction normal to the Au(111) slab. Details and results are discussed in the main text. The procedure used to compute the lifting force is justified in the Methods section .

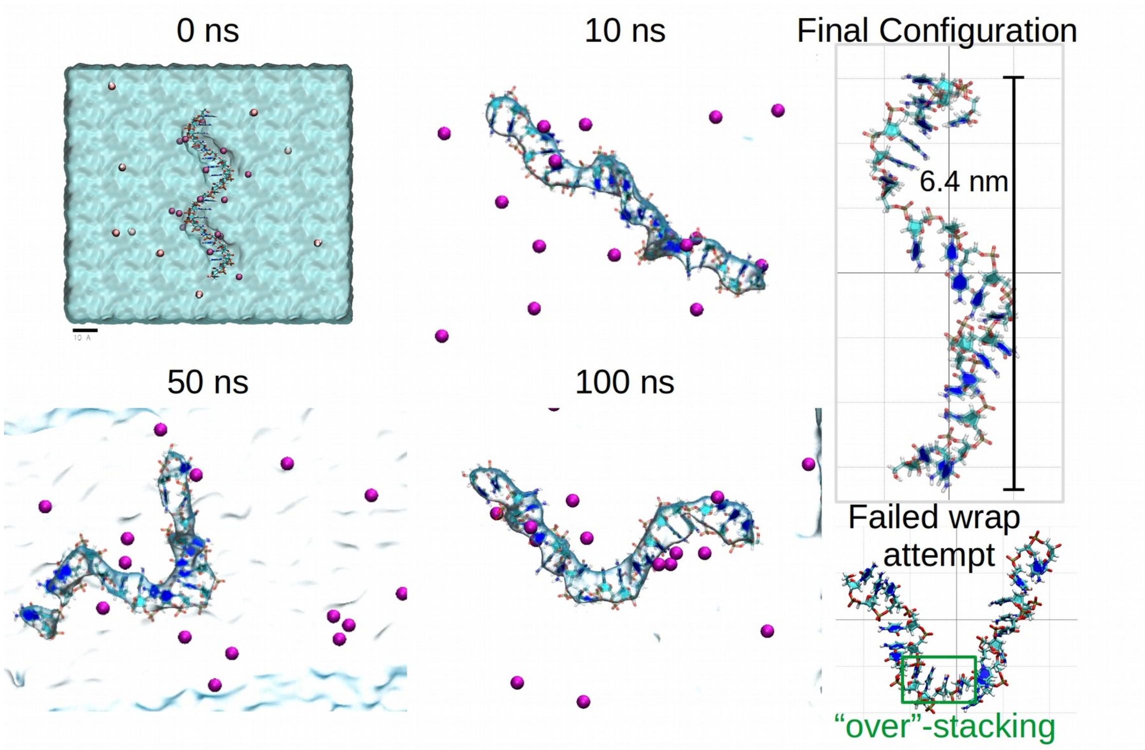

Supplementary Figure 4.100 ns MD simulation of ssDNA fully embedded in water. Side views of oligomer configuration and of charge-compensating Na^+ ions at successive simulation times. Right collumn: favored folded configuration (top) and representative transient configuration (bottom); ions are omitted for

clarity. The top left image is a side view the simulation box.

Supplementary Note 4 : ssDNA structure in water and in nano droplets

In a dilute solution at room temperature a single dsDNA exhibits fluctuating curved conformations under the influence of van der Waals and electrostatic interactions with itself as well as with the surrounding water molecules and ions, e.g. the charge-compensating Na^+ counterions represented by purple VdW spheres in Supplementary Figure 4 and Supplementary Figure 5. Under the same conditions, the same interactions lead to more folded, irregular conformations in the case a single ssDNA, owing to its increased flexibility, stronger π-π stacking interactions and suppressed hydrogen bonding between adjacent bases on the same strand.

To elucidate possible conformational change of the ssDNA oligomer, we performed MD simulations starting from the single-strand canonical B-form of Supplementary Figure 2 fully embedded in water and with all water molecules explicitly included (Supplementary Figure 4). We found that the final configuration deviates from the helical structure of the canonical B-form, albeit without much folding, which we attribute to the limited flexibility of our relatively short oligomer even in the absence of the complementary strand. The stacking between consecutive nucleotide bases is thereby enhanced. Although the molecule tries to bend and wrap in the course of the simulations, these intermediate configurations are transient.

Using the final ssDNA configuration from the previous simulation, we further studied the favored oligomer structure only considering the three first surrounding hydration layers (within ∼1 nm around the oligomer, see Supplementary Figure 5). This ”nano-droplet” containing the ssDNA oligomer and all 19 counter-ions in vacuum (see Supplementary Figure 5 was used for further simulations in the vicinity of the Au surface discussed in the main text. In contrast to ssDNA fully embedded in water (Supplementary Figure 4), the present simulations reveal that the oligomer folds into a compact structure together with the counter-ions within the first 10 ns of the simulation as the droplet size shrinks under surface tension. Afterwards, no major structural changes occur. The final molecule average diameter of ∼3 nm is below the apparent length of adsorbed ssDNA oligomers observed in our STM experiments (see Supplementary Figure 1). Most of the water molecules initially surrounding the oligomer remained within the droplet after the folding until the end of the 100 ns simulation. This indicates that the initial number of water molecules considered is sufficient to hydrate the ssDNA in vacuum conditions.

Supplementary Note 5 : Simulated ssDNA adsorption on Au(111).

We have studied the adsorption of ssDNA on Au(111) using four different simulation protocols. In all cases a similar final conformations were obtained. The first protocol (stage 3) concerned the adsorption of a water droplet containing a ssDNA oligomer. Results are discussed in the main text and all the simulation details are summarized in Supplementary Note 3.

In order to study the influence of water removal on the final adsorption configuration of the oligomer, we performed two additional simulations. In stage

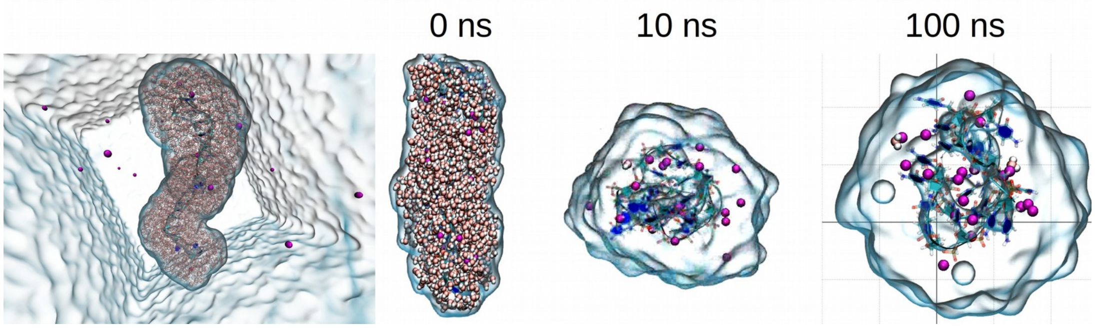

Supplementary Figure 5. 100 ns MD simulation of ssDNA with only its surrounding hydration layer. Side views of oligomer configuration and of Na^+counter-ions. At t=0 the final configuration from the previous simulation together with all water molecules within 1 nm (inside the gray surface in the left image) was relaxed, thus generating the compact nano-droplet bounded by the transparent surface shown at later simulation times.

3a, we anneal up to 450 K the adsorbed droplet obtained at the end of stage 3. The snapshots shown below in Supplementary Figure 6 reveal that during the 100 ns of annealing water starts to evaporate above the adsorbed ssDNA and from the gold surface in its vicinity, whilst keeping the oligomer conformation unaltered. This is compatible with a strong interaction between ssDNA and gold, in particular because most nucleotide bases are in direct contact with the gold surface already prior to annealing, as described in the main text. Since we are performing our MD simulations in the NVT ensemble, the total number of molecules and ions in the system remains constant. The water molecules near the ssDNA are either floating in the simulation box or condensed on the constrained bottom layer of gold in the adjacent box owing to periodic boundary conditions (see Methods).

In stage 3b, we considered same starting adsorbed ssDNA configuration with neutralizing counter-ions but removed all water molecules. Letting the system evolve at 400 K during 100 ns we obtain the final results shown in Supplementary Figure 6 (right) and in greater detail in Supplementary Figure 7b. They clearly show that removing all water molecules has little effect on the final

adsorption configuration of the ssDNA oligomer. We thus conclude that dehydration occurs with little influence on the compact folded conformation of the adsorbed ssDNA oligomer, in particular its width and overall length.

The last adsorption simulation (stage 4) consisted in adsorbing a ssDNA molecule with its neutralizing counter-ions starting from a canonical B-form structure placed 1~nm away from the surface, as shown in Supplementary Figure 7c. Also in this case no water was present. The final results shown in Supplementary Figure 7d confirm that the different adsorption protocols used yield quite similar final configurations, thus explaining why the experimental

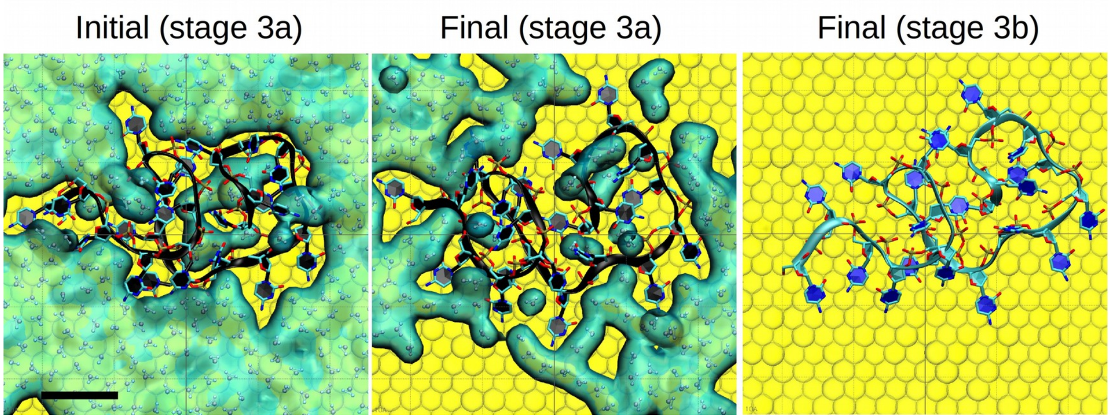

Supplementary Figure 6. Effect of water removal on the final conformation of ssDNA adsorbed on Au(111). From left to right: Initial and final atomic configurations of stage 3a (450 K annealing of the pre-adsorbed droplet) compared to the final configuration obtained using the initially dehydrated adsorbed oligomer (stage 3b). The atomic representation used is the same as in Supplementary Figure 3. Scale bar = 1 nm.

results following dehydration at 440 K show more or less straight 4 nm long structures like our simulated adsorbed single ssDNA configurations.

These simulations confirm the systematic folding of single ssDNA oligomers adsorbed on the gold surface independent of the initial configuration prior to adsorption as well as its hydration state. Note that only a few bases protrude while the rest lie essentially parallel to the surface at an average distance of 0.27 nm from the Au(111) surface layer. Similar adsorption geometries were predicted for an isolated cytosine base in vacuum and in water at 300 K by using a different force field fitted to Van-der-Waals density functional calculations by the same group. The height variation of the adsorbed oligomer is larger than the STM topographic profile in Supplementary Figure 1c , as expected for a poorly conducting molecule.

Supplementary Note 6 : ssDNA diffusion and self-asssembly on Au(111).

To understand the experimentally observed diffusion and self-assembly of ssDNA adsorbed on gold, we performed 500 ns MD simulations of two oligomers at 400 K and 500 K (see stage 5 of Supplementary Note 3). The initial separation of 3nm, corresponds to the average distance between ssDNA molecules observed experimentally at 440 K (see Supplementary Figure 1 b).

Intermediate configurations, as well as the final ones are shown in Supplementary Figure 8. At 400 K, no major structural rearrangements and no diffusion occurred during the 500 ns. In contrast, diffusion is clearly observed at 500 K, thus favoring self-assembly driven by increasing intermolecular interactions and self-assembly. Interestingly the structures of the ssDNA oligomers assembled at 500 K are slightly flattened compared to lower annealing temperatures. Nevertheless, their overall disordered folded configurations remain quite similar to those obtained after deposition of a water nanodroplet at 300 K (Fig. 2a of the main text) or after adsorption and annealing in vacuum at 400 K (Supplementary Figures 7 b and d).

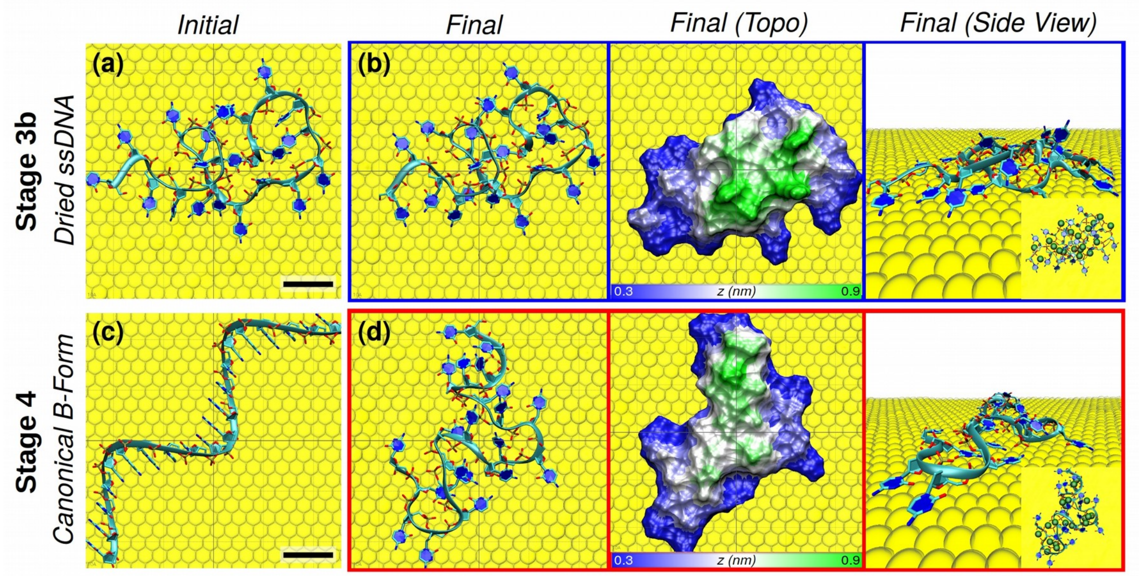

Supplementary Figure 7 MD simulations of single–strand DNA adsorption on Au(111) in vacuum. **a-b,** Initial and final configurations of the ssDNA oligomer pre-adsorbed on gold in a 100-ns MD simulation of annealing at 400 K in vacuum without considering water molecules (stage 3b in Supplementary Note 3). **c-d,** Initial and final configuration starting from a canonical B-form strand in a 100-ns MD simulation of its adsorption on gold at 400 K (stage 4 in Supplementary Note 3). Note that all 19 Na^+ counter-ions were included (shown by green dots in the inset side views on the right). Scale bars=1 nm.

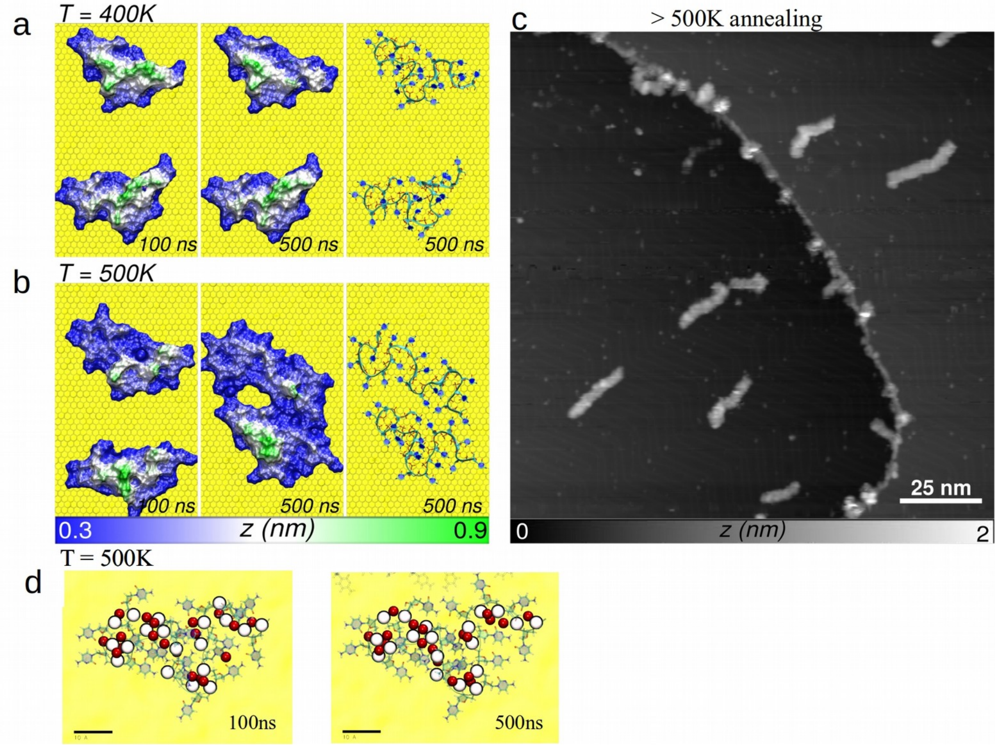

Supplementary Figure 8. Thermally-Activated Diffusion of ssDNA Molecules on Au(111). **a,b,** 500-ns MD simulations of the diffusion of two ssDNA oligomers at 400 K and 500 K. Their initial configurations were the same as in Supplementary Figure 7d. The representations used here are the same as those in the previous figures. At 500 K, the oligomers start diffusing, thus leading to self-assembly driven by intermolecular interactions. **c,** STM topography image of ssDNA oligomers self-assembled at 500K subsequently imaged at 4K (*I* =2 pA, *V* = -1.3 V). **d,** Position of the P atoms (van der Waals spheres) and the Na^+ ions (red spheres) at different stages of the 500 K simulation.

In our experiments (see Supplementary Figure 1), we observed molecular assemblies with lengths over 40nm only after annealing at 500 K. Therefore, and in agreement with our simulations (Supplementary Figure 8), there is no long-range diffusion of dehydrated ssDNA molecules below 500 K. As discussed in the main text, thermal annealing has two direct consequences: water evaporation and ssDNA diffusion. As shown in Supplementary Figure 1, annealing to 440 K results in desorption of water molecules but no long-range diffusion of single ssDNA oligomers identified as the prevalent structures with an overall length ~4 nm matching the simulated ones. Nevertheless, when comparing the STM images after annealing at 340 K and 440 K shown in Supplementary Figure 1a, we observe a higher ssDNA density at 440 K. This difference most likely results

from the uneven surface coverage on larger scales resulting from electrospray deposition. Indeed, before each annealing step, the tip must be retracted thus making it impossible to image the same region of the sample afterwards, owing to thermal drift during annealing.

Although at 300 and 340 K we do not observe long-range diffusion of ssDNA molecules it is instructive to dwell on the possible role of water molecules. Our simulations of hydrated ssDNA (see Supplementary Figure 2 and Supplementary Figure 6) show that the oligomer is in direct contact with the gold surface, i.e. water molecules are only present around or on top of the ssDNA. Based on this direct ssDNA/Au contact, one could argue that water molecules would have a marginal role, if at all, in the diffusion of hydrated ssDNA over that gold surface. In our simulations, we observed that hydrated ssDNA did neither diffuse at 300 K (stage 3) nor even during water evaporation (stage 3a).

Finally, in all MD simulations we found that after adsorption Na^+ counter-ions preserved their relative positions with respect to the ssDNA backbone. Interestingly, this behavior persisted even at 500 K (see Supplementary Figure 8d) when both molecules slightly change their conformations while diffusing. Concurrently, in the experiments we observe that after 440 K annealing the prevalent features display a similar length of about 4nm (see Figure 1a of the main manuscript and Supplementary Figure 1). This observation supports our assumption that they correspond to dehydrated ssDNA molecules with the same number of neutralizing ions. Indeed, a charge undercompensated ssDNA would adopt a more extended conformation to minimize the Coulomb repulsion between uncompensated negative phosphate groups.

Supplementary Note 7 : Lifting experiments on single-ssDNA oligomers.

Single ssDNA oligomers were picked up by first slightly indenting the conducting tip into the gold substrate, then gently pressing it against one end of a selected oligomer, as previously done with single aromatic molecules adsorbed on Au(111) [3], as well as polymers prepared by annealing aromatic precursors on the same surface [4].

Supplementary Figure 9 shows typical $I_t(Z)$ (tunneling current) and $\Delta f(Z)$ (frequency shift) traces recorded vs. tip vertical displacement Z in such an experiment for the single oligomer previously identified in Fig. 1b of the main manuscript. Approach and retraction, shown in red and black, respectively, were performed at velocities of about 10 and 30 $pm \cdot s^{-1}$. When the molecule initially visualized by STM (left inset) attaches to the tip during approach, an abrupt jumps occur in both channels (Z is set 0 at that position). Independent on the

initial ssDNA oligomer morphology – (isolated molecule or part of a self-assembled structure), lifting traces never extended beyond 2 nm. In addition, STM images recorded afterwards typically showed the original oligomer back on the surface, usually in a different conformation, like in the right inset of Supplementary Figure 9, implying that the molecule detached from the tip well before it could be completely detached from the surface. The last peak followed by a smooth negative dip in Supplementary Figure 9 is similar to Δ*f*(Z) traces recorded while lifting single PTDCA molecules from Au(111) **[3]**. Such a trace is expected if no elastic instability occurs, i.e. if the tip-molecule bond to is stiffer than the gradient of the force exerted by the substrate. In the present case the reverse seems to hold, namely a cytosine initially picked up smoothly detaches from the tip, a process which can only be described by a realistic instead of a harmonic potential with constant curvature k_{tip}. However, the preceding dips, as well as those in Fig.3b together with the final drop to 0 are rather triggered by sudden jumps from/on the surface and/or the tip broadened by the much slower response times of the detection electronics.

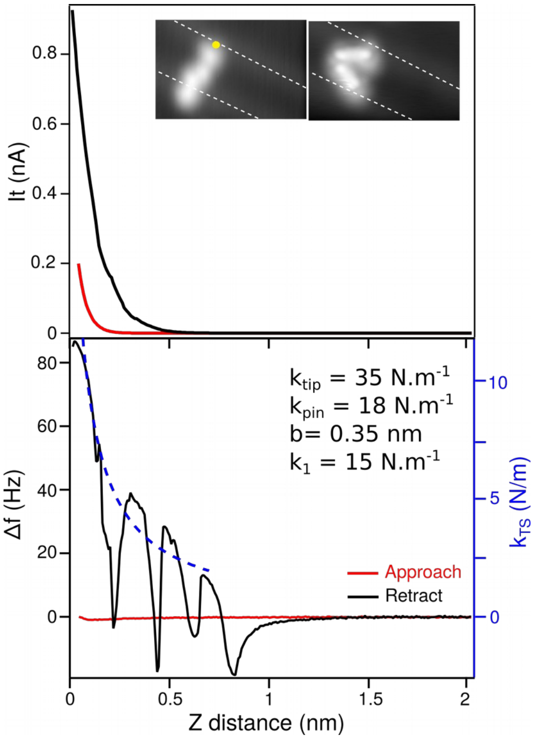

Supplementary Figure 9. Partial lifting of a single ssDNA oligomer. Experimental traces of the tunneling current I_t and the frequency shift $\Delta f \propto k$ simultaneously recorded at 4.8 K on a single oligomer pre-deposited on Au(111) while moving the probing tip towards then away from the gold surface (A =50 pm, f_0 = 26.5 kHz, V = 40 µV). The molecule is attaches to the tip when abrupt jumps are observed in both channels. The blue curve is a tentative fit of k to the springs-in-series model with the indicated parameters. Inset: STM images recorded before and after the manipulation (I =2 pA, V = -1.3 V); the yellow dot indicates the approach point while the dotted lines highlight adjacent crests of the Au(111) herringbone reconstruction.

Supplementary Note 8 : Fitting lifting simulations and experiments to the spring-in-series model. Supplementary Figure 10 shows results of fits to the

maximum force gradients. Like Fig. 3d of the main text, the black traces in Supplementary Figure 10a were obtained by differentiating a narrow (20 pm) running average of the forces *F*(Z) versus vertical tip-surface distance from two independent steered MD simulations for a lower and a much higher k_{tip} value. Least-square fits of Eq.(1) in the main text to maximum $k = dF/dZ$ values between Z = 3 and 11 nm are shown by colored curves together with the respective fit parameters. Resulting *1/k* plots for all three investigated k_{tip} values

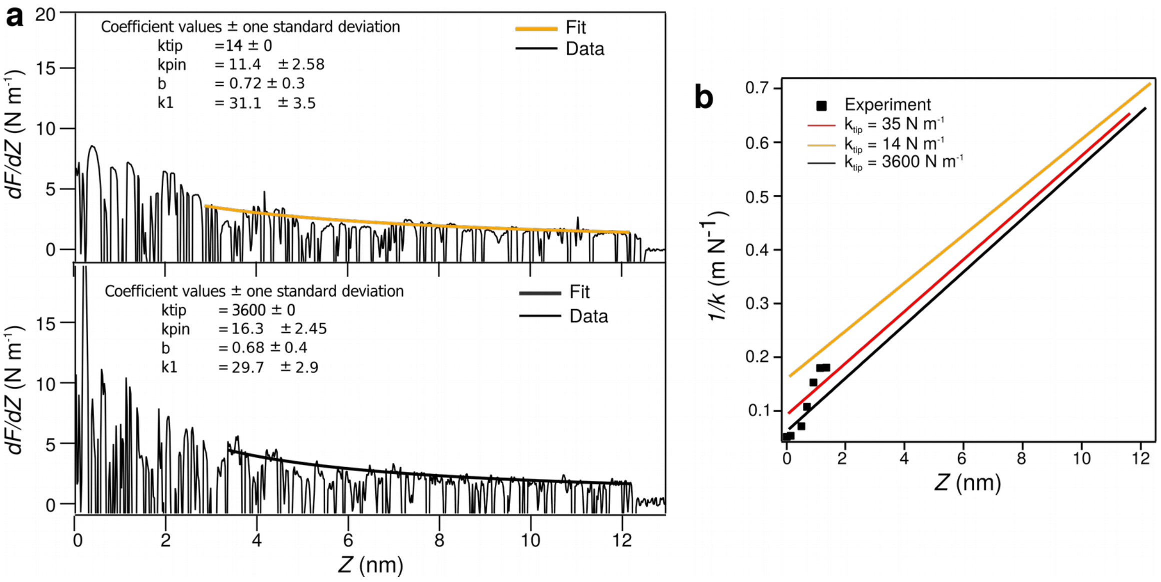

Supplementary Figure 10. Fits to maximum force gradients from simulated and measured ssDNA oligomer lifting traces. **a,** $k = dF/dZ$ traces versus tip atom position Z extracted from computed pulling forces for k_{tip} = 14 (top) and 3600 $N.m^{-1}$ (bottom), together with smooth least-squares fits to the positive gradient maxima. Fit parameters of the springs-in-series model (see main text) are listed above. **b,** Comparison of *1/k*(Z) fits to independent steered MD simulations for k_{tip} = 14, 35 and 3600 $N.m^{-1}$ together with experimental maximum *k*-values (blue) extracted from Fig. 3b.

are shown by the lines in Supplementary Figure 10b together with average *1/k* points on the maxima of the experimental trace in Fig. 3b of the main text. The computed slopes match reasonably well and reveal the stiffness k_1 per repeat unit of fully stretched ssDNA. The experimental points follow the same slope initially but then deviate, thus indicating a strong decrease of *k* which we attribute to a weakening of the interaction between the lifted part and the tip. The description by a constant k_{tip} is only valid when the molecule-tip bond remains

intact. The same applies to the interaction between the lifted and remaining oligomer segments. Thus k_{pin} describes the connection between the P atom about to be detached and the remaining segment attached to the substrate. The adequate description of maximum force gradients by constant k_{pin} values over a wide Z range indicates that individual bases are adsorbed so strongly that the remaining ones are unaffected until they begin to peel off. Over the first 5 nm, the fits lie below the strongest maxima since the first two bases are lifted almost together, and later because the backbone must unwind until it straightens. All those features are vividly illustrated in the attached movies. Fitted b values are close to each other and consistent with simulated detachment distances Z_{off} ~ 12 nm estimated as $Z_{off} = 18.5\, b \cos(\Theta)$ namely b = 0.685 nm. Note finally that our fitted k_1 values are of the same order as the value (17.5 N m^{-1}) calculated for small molecules mimicking the ssDNA backbone using Eq. (2) and Table I in ref.[5] i.e. $F = (\gamma_1 u + \gamma_2 u^2)$ with $b_{0\,=}$ 0.7 nm, γ_1 = 8.44 nN and γ_2 = 29.5 nN), $u = (b - b_0)/b_0 = 0.15$ being the strain calculated for a representative 2.0 nN tensile load.

Supplementary References

[1] Satterley, C. J. *et al.*. Electrospray deposition of fullerenes in ultra-high-cacuum: in situ scanning tunneling microscopy and photoemission spectroscopy. *Nanotechnology* **18,** 455304 (2007).

[2] Kebarle, P. & Verkerk, U. H. Electrospray: from ions in solution to ions in the gas phase, what we know now. *Mass Spectrom. Rev.* **28,** 898-917 (2003).

[3] Wagner, C., Fournier, N., Tautz, F. S., & Temirov, T. Measurement of the binding energies of the organic-metal perylene tetracarboxylic dianhydride/Au(111) bonds by molecular manipulation using an atomic force microscope. *Phys. Rev. Lett.* **109,** 076102 (2012).

[4] Lafferentz, L., Ample, F., Yu, H., Hecht, S., Joachim, C. & Grill L. Conductance of a single conjugated polymer as a continuous function of its length. *Science* **323**, 1193 (2009).

[5] Lipfert, Jan, Doniach, Sebastian, Das, Rhiju & Herschlag, Daniel. Understanding nucleic acid–Ion interactions. *Annu. Rev. Biochem.* **83,** 813-841 (2014).
