# Supplementary material for: Conformations and cryo-force spectroscopy of spray-deposited single-strand DNA on gold
Source: Nat Commun. 2019 Feb 8;10:685. doi: 10.1038/s41467-019-08531-4 (PMC6368621; doi:10.1038/s41467-019-08531-4)
Supplement: Supplementary file 3 — Description of Additional Supplementary Files [file 41467_2019_8531_MOESM3_ESM.docx]

**Description of Supplementary Files**

**File Name:** Supplementary Movie 1

**Description:** Side-views of the simulated lifting process of a 20--cytosine ssDNA oligomer pre-adsorbed on the (unreconstructed) Au111 surface. The ssDNA backbone was fitted by a smooth spline which is represented as a purple tube. Only the cytosine bases and the attached deoxy-ribose atoms (without hydrogens) are shown using a stick representation in order to focus attention on the motion of the most important elements. The bases and riboses are colored accordingly to their proximity to the surface, namely red, green and blue below distances of 0.2, 0.3 and 0.4 nm from the top gold layer. A square grid is superposed with black lines and thin dotted lines separated by 1 nm and 0.1 nm, respectively. The lifting axis passing through the lifted phosphorous atom between the first two nucleotides is represented by the thick vertical dashed line.

**File Name:** Supplementary Movie 2

**Description:** Top-views of the simulated lifting process of a 20-cytosine ssDNA oligomer pre-adsorbed on the Au111 surface. The representation used is the same as in the Side-view movie. The gold atom closest to the lifting axis is highlighted in red.
